# The Rumen Specific Bacteriome in Dry Dairy Cows and Its Possible Relationship with Phenotypes

**DOI:** 10.3390/ani10101791

**Published:** 2020-10-01

**Authors:** Shih-Te Chuang, Shang-Tse Ho, Po-Wen Tu, Kuan-Yi Li, Yu-Lun Kuo, Jia-Shian Shiu, Sheng-Yao Wang, Ming-Ju Chen

**Affiliations:** 1 Department of Veterinary Medicine, National Chung Hsing University, Taichung 402, Taiwan; stchuang@dragon.nchu.edu.tw; 2 Department of Animal Science and Technology, National Taiwan University, Taipei 106, Taiwan; stho@mail.ncyu.edu.tw (S.-T.H.); ray0983631720@gmail.com (P.-W.T.); kyli1022@ntu.edu.tw (K.-Y.L.); yaoyao@ntu.edu.tw (S.-Y.W.); 3 Department of Wood Based Materials and Design, National Chiayi University, Chiayi 600, Taiwan; 4 Biotools Co., Ltd., New Taipei City 221, Taiwan; chris@toolsbiotech.com; 5 Hengchun Branch, Livestock Research Institute, Council of Agriculture, Executive Yuan, Pingtung 946, Taiwan; Wadeshiu@mail.tlri.gov.tw

**Keywords:** dry period, rumen microbiota, physiological roles, *Prevotella*, *Ruminococcus*

## Abstract

**Simple Summary:**

The aim of this study was to investigate the importance of the relationships among hosts, microbiota, and diet composition in dry dairy cows. Therefore, the composition of the rumen microbiome in cows from three dairy farms was investigated to identify core bacteria contributing to various physiological roles during rumen fermentation in dry dairy cows. Our results revealed that the ruminal fluid in dry dairy cows from different regional farms had core rumen microbiota that could be clearly distinguished from that of cows of the other farms.

**Abstract:**

Most microbiome studies of dairy cows have investigated the compositions and functions of rumen microbial communities in lactating dairy cows. The importance of the relationships among hosts, microbiota, diet composition, and milk production remains unknown in dry dairy cows. Thus, in the present study, the composition of the rumen microbiome in cows from three dairy farms was investigated to identify core bacteria contributing to various physiological roles during rumen fermentation in dry dairy cows. The results indicated that ruminal fluid in dry dairy cows from different regional farms had core rumen microbiota that could be clearly distinguished from that of cows of the other farms. Further identification of key microorganisms associated with each farm revealed that *Prevotella*, *Methanobrevibacter*, *Pseudobutyrivibrio*, *Ruminococcus*, *Bacteroides*, and *Streptococcus* were major contributors. Spearman’s correlation indicated that the abundance of genera such as *Prevotella* and *Ruminococcus* in dry dairy cows could indicate milk yield in the previous lactating period. Functional pathway analysis of the rumen bacterial communities demonstrated that amino acid metabolism and carbohydrate metabolism were the major pathways. Our findings provide knowledge of the composition and predicted functions of rumen microbiota in dry dairy cows from regional farms, which underscore the importance of the relationships among hosts, microbiota, diet composition, and milk production.

## 1. Background

The rumen, the forestomach of ruminants, harbors bacteria, archaea, fungi, and ciliate protozoa [1]. An indispensable function of these microorganisms is to break down plant polymers into volatile fatty acids through various fermentation pathways to be absorbed and used by host animals [1,2]. The composition and function of rumen microbial communities are not only affecting growth and milk production but are also related to the host health and nutrient utilization in dairy cattle [3,4]. The unique metabolites (e.g., saturated fatty acids, organic acids, amine, or polysaccharide) produced by rumen microbes may play a vital role in ruminant physiology [5]. Jewell et al. [6] reported that some rumen bacteria are associated with milk production efficiency and ketosis.

Many factors, including diet, species, age, and geographic location, can affect the composition and function of rumen microbiota [6,7]. Furthermore, the acidosis, subacidosis, and metabolic dysfunction of the rumen can change the composition and function of rumen microbiota [8,9,10]. Given the importance of rumen microbial communities on ruminant health and productivity, many researchers have attempted to manipulate rumen microbiota through various strategies, such as using chemical agents and enzymes as feed additives or probiotic supplements. However, modification of rumen microbial compositions in adult ruminants appears difficult. Changes in rumen microbiota from exogenous factors can be rapidly restored by eliminating the influential factors [11], but little is known of the ecological and physiological roles of predominant core bacteria in the rumen microbial ecosystem.

In addition, the lactation cycle is split into lactation and dry periods. Most studies have focused on investigating rumen microbiota during lactation; for instance, studies have assessed how rumen microbiota respond to exogenous butyrate [12] and the composition of rumen microbiota [13,14]. The dry period is a crucial rest period for cows in which new hormonal stimulation is gained for subsequent lactation. The precise interaction between a rumen microbial community and the host animal should be investigated for the whole lactation cycle to investigate the possibility of maintaining the health and productivity of ruminants by manipulating rumen microbial composition. Nevertheless, limited information has been reported concerning rumen microbiomes and their functions during dry periods.

Thus, in the present study, the composition of rumen microbiomes from three dairy farms in the northern, middle, and southern regions of Taiwan, respectively, were investigated. We identified core bacteria that contribute to various physiological roles during rumen fermentation in dry cows. The exogenous factors affecting rumen microbiomes were also studied. Fundamental knowledge of rumen core microbiomes and their relationship to physiological functionality in rumen microbial ecosystems during dry periods can provide insight into potential manipulation of rumen microbiota to enhance dairy performance, such as milk production and cow health.

## 2. Methods

All experimental procedures were conducted according to guidelines from the Institutional Animal Care and Use Committee of National Taiwan University (IACUC number: NTU105-EL-00022). The owners’ consent was verbally confirmed as part of routine veterinary operation.

### 2.1. Animal Sources and Sampling

All experimental procedures were conducted according to guidelines from the Institutional Animal Care and Use Committee of National Taiwan University (IACUC number: NTU105-EL-00022). In total, 15 Holstein cows in the second week of their dry periods from three farms were sampled in Taipei (experimental farm of the National Taiwan University, Farm A, *N* = 5), Changhua (private farm, Farm B, *N* = 5), and Tainan (Lin Fengying’s daily farm, Farm C, *N* = 5). The animals were fed with a mixed forage–concentrate diet with different diet compositions among the farms (Farm A: 10.71% crude protein (CP) and 60.90% crude fiber (CF); Farm B: 9.92% CP and 66.79% CF; Farm C: 13.20% CP and 43.08% CF). The forage-to-concentrate ratios (F:C) in diet dry matter (DM) basis of Farms A, B and C were 85.7:14:3, 88.8:11.2, and 67.5:32.5, respectively (Table 1). Average milk production during the previous lactating period for the cows of Farms A, B, and C were 24.1 ± 8.3, 22.5 ± 7.6, and 20.1 ± 8.5 kg, respectively. The cows’ entire mid-rumen fluid contents (approximately 200 mL) were collected using a stainless-steel stomach tube by a veterinarian, and their entire rumen fluid volume was filtered with gauze to remove solids. After this study, all experimental animals were released and then maintained by the original farms.

### 2.2. DNA Extraction and Next-Generation Sequencing

Rumen samples were immediately frozen in a −80 °C freezer and then freeze-dried. DNA was extracted from 100 mg of homogenized rumen sample through bead beating followed by phenol–chloroform extraction [5]. The resulting solution was precipitated with ice-cold isopropanol (Sigma-Aldrich, St. Louis, MO, USA) to obtain the DNA pellet. The pellet was washed with 70% ethanol twice and suspended in 200 μL of ddH_2_O. The DNA was quantified using a Nanodrop 2000 spectrophotometer (Thermo Scientific, Waltham, MA, USA) and stored in a −20 °C freezer until analyzed. For next-generation sequencing (NGS), the 16S ribosomal RNA (rRNA) gene regions were amplified for further analysis. The primer set used in this study was 16S, 515F, and 806R for amplification of the V4 16S rRNA gene region. The PCR reaction was conducted in a PCR machine (Biometra T3000 thermocycler, Analytik Jena, Göttingen, Germany); the PCR conditions are shown in Supplementary Materials Table S1.

### 2.3. Bioinformatics

Amplicon sequencing was performed using the paired-end Illumina HiSeq platform to generate 250 bp paired-end raw reads, and the entire set of paired-end reads was assembled using FLASH v.1.2.7 [15]. Demultiplexing was conducted from barcode identification. For quality control, low-quality reads (Q < 20) were discarded in the QIIME 1.9.1 pipeline [16]. If three consecutive bases were Q < 20, the read was truncated and retained in the dataset only if it was at least 75% of the original length; this was done using the split_libraries_fastq.py script in QIIME [17]. Sequences were chimera-checked using UCHIME to obtain effective tags and were filtered from the dataset before operational taxonomic unit (OTU) clustering at 97% sequence identity using the UPARSE function in the USEARCH v.7 pipeline [18,19,20,21]. For each representative sequence, RDP Classifier v.2.2 [22] was employed to annotate taxonomy classification based on the information retrieved from the SILVA database v.132 [23,24]. This was performed with a minimum confidence (80%) threshold to record assignments. Sequences with one-time occurrence (singletons) or that were present in only one sample were filtered out. To analyze sequence similarities among OTUs, multiple sequence alignment was conducted with PyNAST v.1.2 [25] against the core-set dataset of the SILVA database. A phylogenetic tree was constructed with sequences representative of the OTUs using FastTree [26,27].

To normalize varying sequence depths across samples, OTU abundance information was rarefied to the minimum sequence depth using the QIIME script (single_rarefaction.py). Subsequent analysis of alpha and beta diversity was performed using normalized data. Alpha diversity indicated species complexity within individual samples from seven criteria outputs from the QIIME pipeline, including observed OTUs, Chao1, ACE (abundance-based coverage estimators), Shannon, Simpson, phylogenetic diversity whole tree, and Good’s coverage [28]. Observed OTUs signified the number of species represented in a microbial community. Community richness was assessed using Chao1 and ACE indices, and the relative abundance and evenness accounting for diversity were evaluated with Shannon and Simpson indices. A rarefaction curve was constructed from random selection of sequencing data from each sample to represent the number of observed species [29].

A distance matrix of unweighted UniFrac and Bray–Curtis dissimilarity among previously obtained samples was transformed into a new set of orthogonal axes, where the most influential variable was represented by the first principal coordinate and the second most influential variable by the second principal coordinate, and so on. An unweighted pair-group method with arithmetic mean hierarchical clustering was performed to interpret arithmetic distances with an average linkage algorithm. For statistical analysis, a Mann–Whitney U test was used to determine the significance of alpha diversity using R software. For beta diversity, principle components analysis (PCA) was performed to evaluate differences between sample groups. Spearman correlation analysis was applied to measure co-occurring correlation of the top 15 genera (the most abundant genera). Statistically significant biomarkers were identified through linear discriminant analysis (LDA) effect size (LEfSe), which is an approach based on an algorithm that performs nonparametric Kruskal–Wallis and Wilcoxon rank-sum tests to identify bacterial taxa with significantly different abundance between each group [30]. LEfSe applies LDA to bacterial taxa identified as significantly different and assesses the effect size of each differentially abundant taxon. In this study, taxa with LDA scores (log 10) > 4 were considered significantly different. For functional analysis, the functional abundance of 16S rRNA sequencing data was analyzed to predict functional genes with PICRUSt v.1.1.1 [31].

## 3. Results

### 3.1. Proportion of Rumen Bacterial Communities in Dry Cows Was Diverse Across Regional Farms

**Figure 1:**
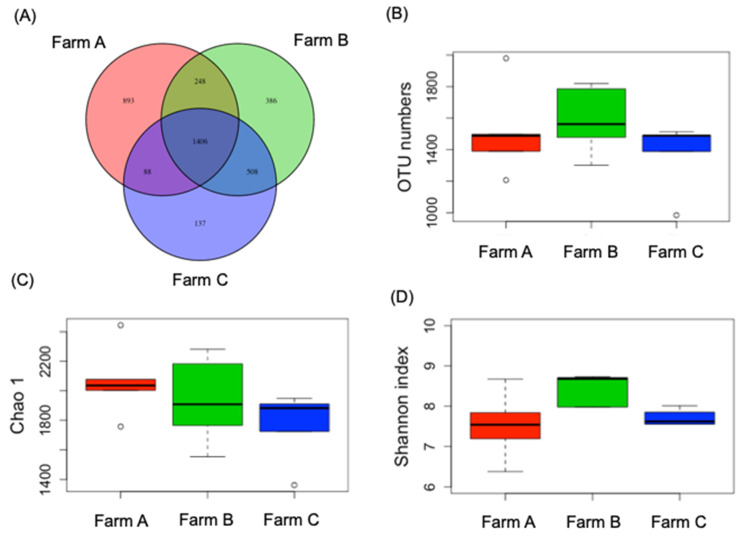
Venn diagram (**A**), operational taxonomic unit (OTU) numbers (**B**), Chao1 values (**C**), and Shannon index (**D**) of cow rumen microbiota from regional farms.

First, we examined the rumen microbiota of the dry cows from the various farms using NGS. The results indicated that 24,390 valid sequences of bacterial 16S rRNA genes were obtained for analysis. As presented in Figure 1A, 1406 OTUs in all samples were shared among the farms, with 893, 386, and 137 unique OTUs for Farms A, B, and C, respectively. The observed OTUs of Farms A, B, and C numbered 1589.2 ± 193.9, 1512.2 ± 255.6, and 1373.3 ± 198.9, respectively, with no significant difference between the farms (Figure 1B). For α-diversity in each group, the Chao1 (richness) and Shannon indices (diversity) of Farms A, B, and C were not significantly different (Figure 1C,D).

**Figure 2:**
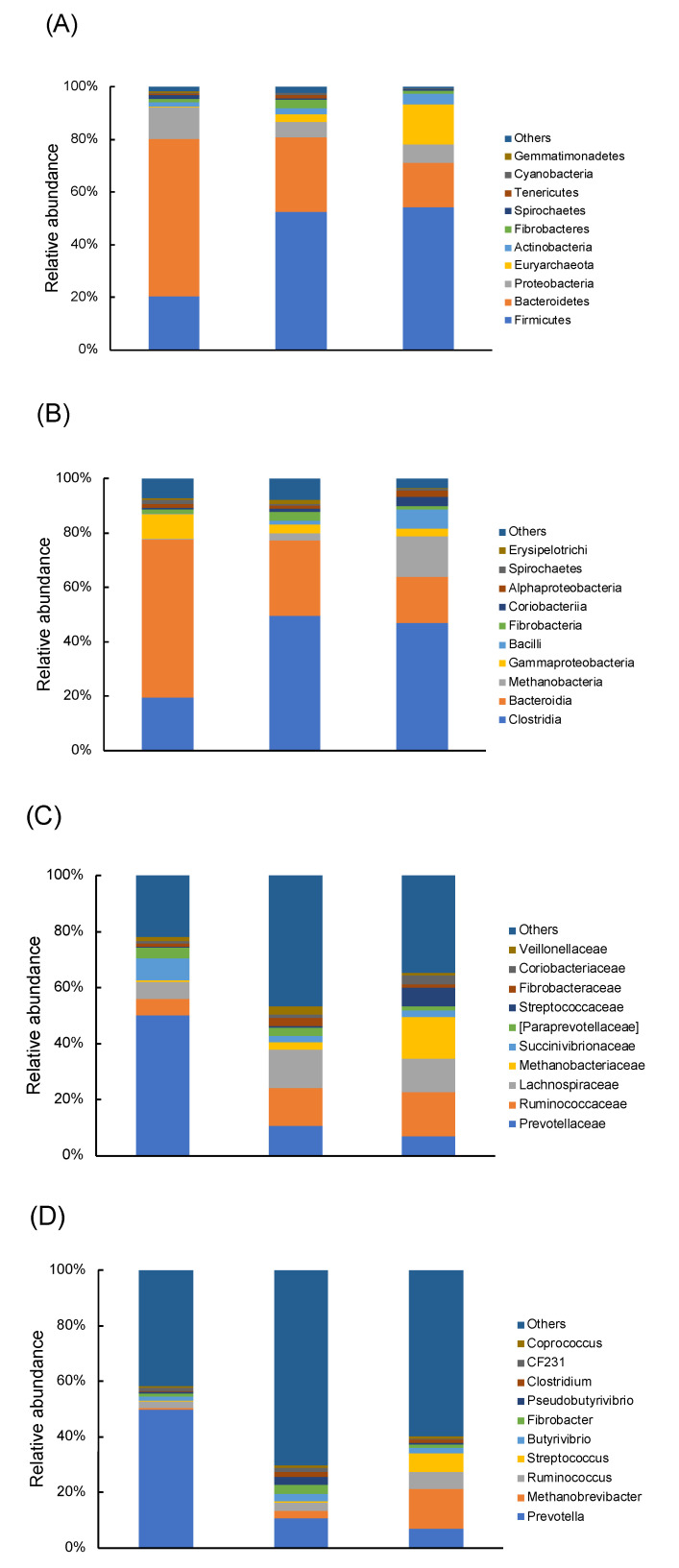
Relative abundance of predominant rumen microorganism populations at phylum (**A**), class (**B**), family (**C**), and genus (**D**) level.

Bacterial taxa were detected in the rumen of all tested cows, with 53 genera belonging to 15 phyla (Figure 2A). These phyla accounted for more than 95% of all bacterial sequences. Fourteen phyla, including Actinobacteria, Bacteroidetes, Cyanobacteria, Elusimicrobia, Euryarchaeota, Fibrobacteres, Firmicutes, Proteobacteria, Spirochaetes, SR1, Synergistetes, Tenericutes, TM7, and Verrucomicrobia, were distributed across all three groups (Farms A, B, and C). The top three dominant bacterial phyla were Firmicutes, Bacteroidetes, and Proteobacteria among the farms, but the proportions of bacterial phyla differed greatly (Figure 2A). Similar patterns were observed in class and family levels (Figure 2B,C). The top 10 dominant bacterial classes and families were the same across groups but with varying proportions. For the genus level, 281 taxa were identified. *Prevotella* occupied 49.9%, 10.8%, and 6.8% of total OTU relative abundance in Farms A, B, and C, respectively, and was the most dominant genus across farms (Figure 2D). Except for *Prevotella*, the abundance of other genera in Farm A was lower than 5%.

### 3.2. Dry Cows from Regional Farms Contained Clearly Recognizable Rumen Microbiota

**Figure 3:**
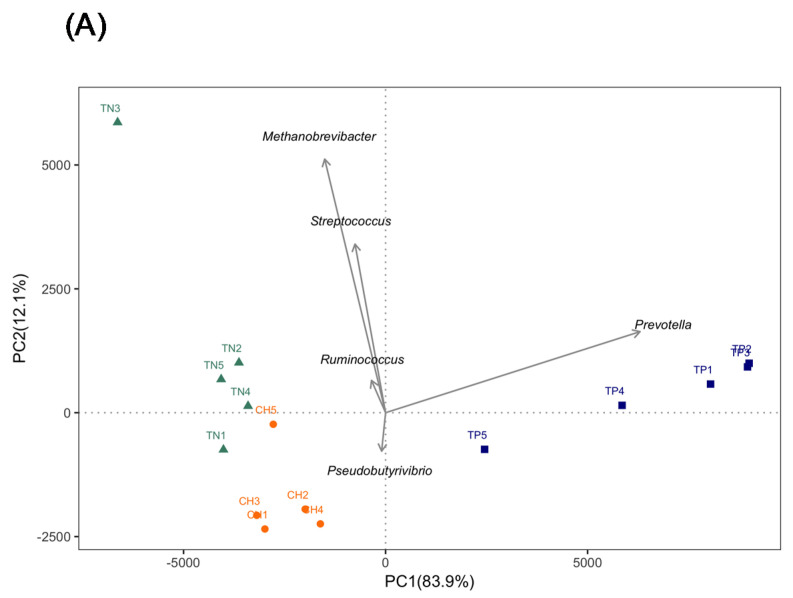

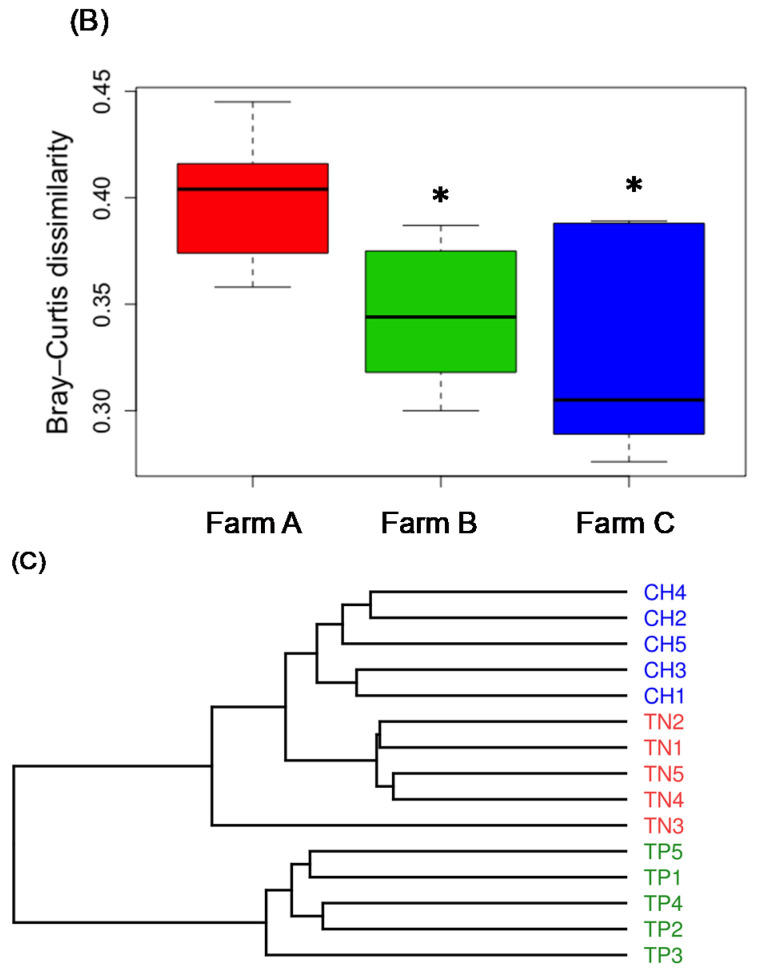
Principal components analysis (**A**), Bray–Curtis dissimilarity (**B**), and unweighted UniFrac (**C**) of cow rumen microbiota from regional farms in Taiwan. * Significant difference (*p* < 0.05) from Farm A. TP = Farm A, CH = Farm B, and TN = Farm C.

We performed PCA on the genus level to evaluate variations in rumen microbial composition among the farms. A PCA plot revealed that PC1 and PC2 accounted for 83.9% and 12.1% of rumen microbiota compositional variation, respectively, and differed among farms (Figure 3A). Vector analysis indicated that *Prevotella* was the major bacteria with higher contributions to PC1 variability. *Methanobrevibacter*, *Pseudobutyrivibrio*, *Ruminococcus*, and *Streptococcus* were major contributors to PC2 variability. Additional analysis with Bray–Curtis dissimilarity and UniFrac revealed results consistent with those of PCA. The regional farms had diverse microbial communities that could be clearly identified between farms (Figure 3B,C).

### 3.3. LEfSe Analysis Identified Biomarkers of Rumen Microbiota from Regional Farms

We identified bacterial taxa that were predominant as biomarkers among the groups through LEfSe. A total of 32 influential taxonomic clades (LDA score > 4) were recognized with six genera biomarkers (Figure S1A,B). The most affected bacterial genus was *Prevotella* in Farm A. In Farm B, the specific biomarker was *Psudobutylvibrio*. Four genera, namely *Bacteroides*, *Methanobrevibacter*, *Ruminococcus*, and *Streptococcus*, were identified as biomarkers in Farm C.

### 3.4. Diet Composition Affected the Network of Co-Occurring Predominant Bacteria at the Genus Level in Rumen

**Figure 4:**
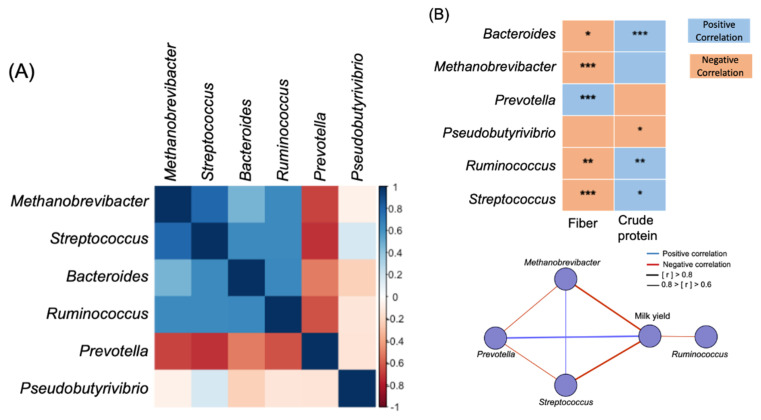

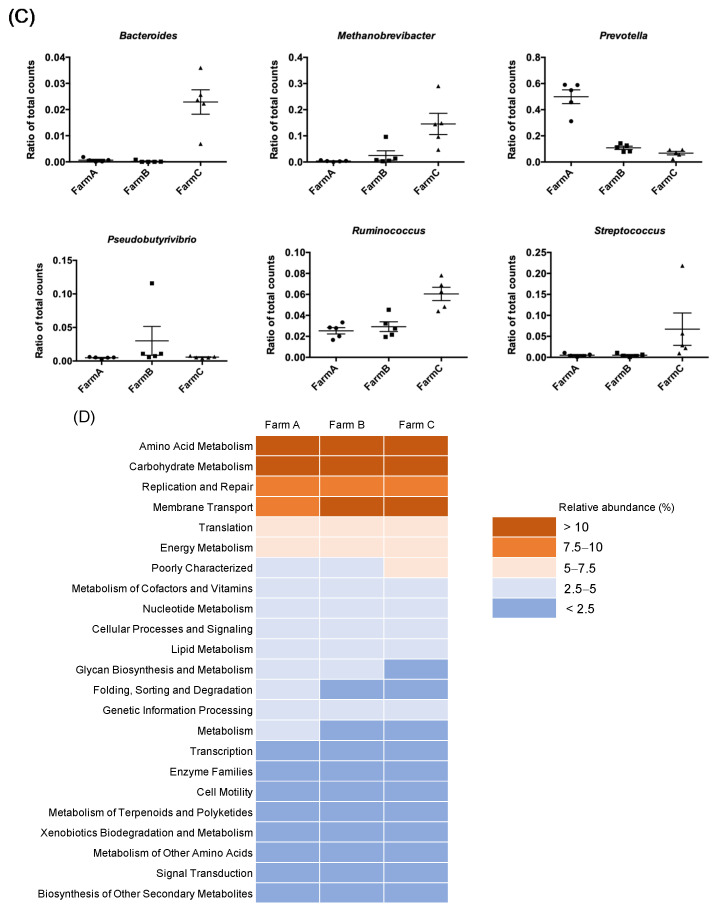
(**A**) Co-occurring analysis of the biomarker genus calculated by Spearman correlation. (**B**) Correlation between diet nutrients/biomarker genus and milk yield/biomarker genus. (**C**) Relative abundance of biomarker genus. (**D**) Abundance of Kyoto Encyclopedia of Genes and Genomes (KEGG) pathways of rumen bacterial communities from regional farms. Only predominant KEGG pathways (relative abundance >1%) are shown. * *p* < 0.05, ** *p* < 0.01, *** *p* < 0.001.

After identifying the bacterial taxa that were predominant as biomarkers among the farms, the roles of these biomarkers in the composition of diet and milk yield from the previous lactating period were investigated. First, a module of microbiome networks among six LEfSe-selected genera were constructed for all cows (Figure 4A,B). *Prevotella* was negatively correlated with *Methanobrevibacter* (*p* = 0.03, r = −0.56), *Bacteroides* (*p* = 0.04, r = −0.54), *Ruminococcus* (*p* = 0.01, r = −0.64), and *Streptococcus* (*p* < 0.01, r = −0.73). Another critical genus, *Ruminococcus*, was positively correlated with *Streptococcus* (*p* < 0.01, r = 0.64), *Methanobrevibacter* (*p* = 0.03, r = −0.56), and *Bacteroides* (*p* = 0.04, r = −0.54). We correlated six LEfSe-selected genera with diet fiber and protein. The genus *Prevotella* (*p* = 0.03, r = −0.56) was positively correlated with diet fiber and negatively correlated with protein, whereas *Methanobrevibacter*, *Bacteroides*, *Ruminococcus*, and *Streptococcus* were negatively correlated with diet fiber (Figure 4A,B). We validated these results by comparing genus-level taxonomic abundance among farms, where we discovered that *Methanobrevibacter*, *Bacteroides*, *Ruminococcus*, and *Streptococcus* were enriched in diets with high protein and low fiber content (Figure 4C). The genera *Methanobrevibacter*, *Ruminococcus*, and *Streptococcus* were negatively correlated with milk yield, whereas *Prevotella* was positively correlated.

### 3.5. Functional Prediction Revealed Similar Physiological Functions of Rumen Microbiota in Dry Cows across Regional Farms

Finally, we evaluated the functional profiles of the rumen microbiota with the Phylogenetic Investigation of Communities by Reconstruction of Unobserved States (PICRUSt) and Kyoto Encyclopedia of Genes and Genomes (KEGG) sequenced database. PICRUSt analysis identified 41 level 2 KEGG pathways in the rumen samples across the regional farms. Among these predictive pathways, the abundance of 17 pathways was higher than 1% (Figure 4D). Among these pathways, the four predominant pathways were amino acid metabolism (10.4% in Farm A, 10.0% in Farm B, 10.0% in Farm C), carbohydrate metabolism (10.3% in Farm A, 10.1% in Farm B, 10.4% in Farm C), replication and repair (9.5% in Farm A, 9.1% in Farm B, 8.7% in Farm C), and membrane transport (9.2% in Farm A, 11.2% in Farm B, 11.3% in Farm C). In addition, translation and energy metabolism were two other critical pathways with relative abundance higher than 5% in the rumen samples (Figure 4D).

## 4. Discussion

Our findings revealed that ruminal fluid in dry dairy cows across regional farms had similar core rumen microbiota but in different proportions. Although bacterial enumeration is difficult to extrapolate from sequencing data, proportional changes within the core microbiota species may be crucial and merit investigation. Some core bacterial taxa identified during the dry period were consistent with those identified in lactating dairy cows [14,32] and beef cattle [33]. The genera *Prevotella*, *Ruminococcus*, and *Butyrivibrio*, which are the most abundant in rumens of lactating dairy cows [14], were the predominant genera in rumens during the dry period. *Prevotella*, from Bacteroidetes, occupies the ecological niche of second line degrader and possesses oligosaccharolytic and xylanolytic activity to produce substantial amounts of succinate and acetate [34]. The genus *Ruminococcus*, which breaks down fibrous plant material to generate acetate, formate, succinate, and other short-chain fatty acids [1], was identified as the second most predominant (8.42%) core taxon in the present study. *Butyrivibrio*, in the class Clostridia, is involved in various ruminal functions, including fiber degradation, protein degradation, lipid biohydrogenation [35], and microbial inhibitor production [36,37]. Mrázek et al. [38] reported that high-fiber intake essentially increases *Butyrivibrio* in rumens, whereas high-energy food additives suppress it. This genus also significantly differed among somatic cell count groups [39]. Our study was paralleled with previous findings in *Butyrivibrio*. Farm B, with a higher fiber intake than the other two farms, showed more abundance in *Butyrivibrio* (Farm A: 1.5 ± 0.2%, Farm B: 2.7 ± 1.3%, Farm C: 2.0 ± 0.6%). Although ruminal fluid in dry dairy cows across regional farms possessed similar core rumen microbiota, variation in rumen microbiota composition could effectively separate each farm by PCA plot and unweighted UniFrac. *Prevotella*, *Methanobrevibacter*, *Pseudobutyrivibrio*, *Ruminococcus*, *Bacteroides*, and *Streptococcus* were major contributors in this respect. Variations in identified core rumen microbiomes in dry dairy cows may be attributed to differences in dietary conditions (forage-to-concentrate ratio), geographical location, and management regime. Indugu et al. [40] indicated that differences in microbial communities between farms are greater than within farms, which is similar to our findings.

Factors affecting specific bacterial genera must be evaluated. We identified key microorganisms associated with each farm using LEfSe. The results agreed with our PCA findings. Through additional Spearman correlations, the genus *Prevotella*, a biomarker in Farm A, was positively correlated with milk yield in the previous lactating period. Studies have indicated that ruminal *Prevotella,* which can convert sugars, amino acids, and peptides into energy [41,42], was significantly higher in high milk yielding cows as compared to the low milk yielding cows [40]; this association has been shown for both high- and low-milk yielding cows [6]. Moreover, the relative abundance of short-chain fatty acid (SCFA)-producing genera, including *Prevotella* and other genera (*Bacteroides*, *Oscillibacter*, *Clostridium*, *Succinivibrio*, and *Phascolarctobacterium*), among the identified core ruminal microorganisms in Farm A comprised more than 25% of the total sequences in our dataset, which was higher than that of the other two farms. SCFAs serves as an important energy source for epithelial cells in ruminants [43] and are significant in maintaining colonic health in both humans and animals [44].Sufficient energy is required to maintain cow health and support a high milk yield because gut SCFAs are precursors to milk fats [45]. The relative abundance of SCFA-producing genera may partially explain the higher milk yield of Farm A in the previous lactating period. The results revealed that the abundance of *Prevotella* with SCFA-producing microorganisms in dry dairy cows may indicate milk yield during the previous lactating period.

*Pseudobutyrivibrio*, the specific biomarker from Farm B, was positively correlated with fiber and negatively correlated with milk yield. *Pseudobutyrivibrio*, a Gram-negative, anaerobic, and non-spore-forming bacterial genus from the Lachnospiraceae family, was reported to have a functional role in the digestion of hemicellulose [46]. The dry cows from Farm B were fed 80% from pastures, and cows from Farms A and C were fed 70% and 60% from pastures, respectively. Therefore, the dry cows from Farm B had a higher relative abundance of *Pseudobutyrivibrio*.

In Farm C, the specific biomarkers were the genera *Methanobrevibacter*, *Ruminococcus*, *Bacteroides*, and *Streptococcus*, which were all negatively related to fiber in the present study. *Methanobrevibacter* is in the Methanobacteriaceae family. Certain *Methanobrevibacter* groups of *Methanobrevibacter* species, including *M. smithii*, *M. gottschalkii*, *M. millerae*, and *M. thaueri*, were correlated with individuals with higher CH_4_ production [47,48], with no effect on fiber digestion or milk production [49]. *Ruminococcus*, a major fiber and cellulose degrader in the rumen of ruminants [50], was negatively correlated with fiber and milk yield in the present study. Jami et al. [13] reported that *Ruminococcus* was negatively correlated with milk production, whereas *Streptococcus* was positively correlated with starchy diets. In a study, *Bacteroides* was found to be significantly reduced in abundance in rumen fluids because of some diseases [51]. The feeding of dairy cows with probiotics in Farm C may have resulted in significant increases in rumen fermentative bacteria (including *Bacteroides* and *Ruminococcus*), which corresponds with a previous study [52]. In addition, introducing other microorganisms, such as probiotics, can modulate gut SCFAs by changing the metabolism of certain intestinal microflora during ruminal fermentation [53]. In the present study, only Farm C provided probiotics to dry dairy cows, which did not reveal more SCFA-producing microorganisms. Weimer [11] reported that rumen microbiomes exhibit remarkable specificity and resilience within hosts. Most attempts at introducing probiotics to rumens have resulted in only a temporary change after days or a few weeks, suggesting high host-specificity of rumen microbiome composition once established [54].

The similarity of core bacteria was associated with the major metabolic pathways, and functional pathway analysis of rumen bacterial communities unsurprisingly revealed that amino acid and carbohydrate metabolisms were the major pathways. Moreover, the consistent presence of core taxa in the rumen indicated the vital functions of rumen ecological niches in dairy cows [14]. A metaproteomics study agreed with our findings regarding relative bacterial abundance, reporting that Bacteriodete activity dominated the metaproteome most abundantly with the Prevotellaecae family [55]. *Prevotella* are typically related to microbial proteolytic activity in the rumen [56] and early colonization associated with fiber degradation [57]. Degradation of fiber through fibrolytic bacteria activity is crucial for rumen microbiota to gain energy [58]. Once thought to be abundant in the rumen, *Ruminococcus*, the second most dominant genus in rumen bacterial communities, contributes to the degradation of plant polymers, which suggests that this genus plays a key role in carbohydrate metabolism. However, in the present study, we were unable to clarify which factor (regional difference or diet composition) was more effective on the change of rumen microbiota in dry dairy cows. Further studies should be conducted in various regional farms with the same diet composition. On the other hand, the present study revealed that several genera were the biomarkers in each farm with a different diet composition. How the diet composition affects the metabolic interaction of those microorganisms in dry cows remains unclear. The metabolomics of dry cows will be investigated in our future study as well.

## 5. Conclusions

In conclusion, unlike other studies that have focused on the lactating period, the present work emphasized the necessity and importance of the relationships among the host, microbiota, and diet composition by analyzing the functions of rumen microbiota in dry dairy cows. The differences among microbial communities across farms were greater than those within farms, mainly because of diet composition. The abundance of certain genera, such as *Prevotella* and *Ruminococcus*, and SCFA-producing microorganisms in dry dairy cows may be related to milk yields from previous lactating periods. Functional analysis of the roles of bacterial communities within the rumen among the farms provided an understanding of the potential for diversified niches and vital functions in the rumen of dry dairy cows. Future studies on other microorganisms, including protozoa, fungi, and archaea, are needed for comprehensive understanding of their roles in dry dairy cows.

## 6. Declarations

### 6.1. Consent for Publication

Not applicable.

### 6.2. Availability of Data and Materials

The datasets used and/or analyzed in the current study are available from the corresponding author on reasonable request.

## Figures and Tables

**Table 1:** The dietary information of dry cows in different farms.

**Farm A**	
Major ingredient	Dry cow concentrate, Bermuda grass
F:C (DM)	85.7:14:3
Crude protein (% DM)	10.71
Crude fiber (% DM)	60.90
Calcium (% DM)	0.60
Phosphorus (% DM)	0.25
**Farm B**	
Major ingredient	Dry cow concentrate, Bermuda grass, Pennisetum grass
F:C (DM)	88.8:11.2
Crude protein (% DM)	9.92
Crude fiber (% DM)	66.79
Calcium (% DM)	0.66
Phosphorus (% DM)	0.34
**Farm C**	
Major ingredient	Dry cow concentrate, Bermuda grass, corn silage, oat grass
F:C ratio	67.5:32.5
Crude protein (% DM)	13.20
Crude fiber (% DM)	43.08
Calcium (% DM)	0.45
Phosphorus (% DM)	0.39

F:C = forage-to-concentrate ratios; DM = dry matter.

## Supplementary Materials

The following are available online at https://www.mdpi.com/2076-2615/10/10/1791/s1, Figure S1: Linear discriminant analysis (LDA) effect size of the OTUs with significant differences in abundance in rumen samples from regional farms. (A) Taxonomic cladogram of 16S rRNA sequences. (B) LDA scores of biomarkers in the rumen samples from different regions The OTUs with LDA scores (log 10) higher than 4 were considered biomarkers for each group. Relative abundance of (C) Bacteroidaceae, Coriobacteriaceae, Lachnospiraceae, Methanobacteriaceae, Paraprevotellaceae, Prevotellaceae, Ruminococcaceae, Streptococcaceae, and Veillonellaceae, Table S1: PCR conditions.

## Abbreviations

ACE	abundance-based coverage estimators
CF	crude fiber
CP	crude protein
KEGG	Kyoto Encyclopedia of Genes and Genome
LDA	linear discriminant analysis
OTU	operational taxonomic unit
PCA	principal components analysis
PICRUSt	phylogenetic investigation of communities by reconstruction of unobserved states
rRNA	ribosomal RNA
SCFA	short-chain fatty acid
