# Supplementary material for: The Rumen Specific Bacteriome in Dry Dairy Cows and Its Possible Relationship with Phenotypes
Source: Animals (Basel). 2020 Oct 1;10(10):1791. doi: 10.3390/ani10101791 (PMC7601041; doi:10.3390/ani10101791)
Supplement: Supplementary file 1 [file animals-10-01791-s001.pdf]

Supporting information

The Rumen Specific Bacteriome in Dry Dairy Cows and Its Possible Relationship with Phenotypes

Shih-Te Chuang [1, #], Shang-Tse Ho [2, 3, #], Po-Wen Tu[2], Kuan-Yi Li[2], Yu-Lun Kuo[4], Jia-Shian Shiu[5], Sheng-Yao Wang[2], and Ming-Ju Chen[2, *]

[1] Department of Veterinary Medicine, National Chung Hsing University, Taichung 402, Taiwan

[2]Department of Animal Science and Technology, National Taiwan University, Taipei 106, Taiwan

[3] Department of Wood Based Materials and Design, National Chiayi University, Chiayi 600, Taiwan

[4] Biotools Co., Ltd, New Taipei City 221, Taiwan

[5] Hengchun Branch, Livestock Research Institute, Council of Agriculture, Executive Yuan, Pingtung 946, Taiwan

[#] These authors contributed equally to this work.

[*]Corresponding author: Ming-Ju Chen

Mailing address: Department of Animal Science and Technology, National Taiwan University, No. 50, Lane 155, Sec. 3. Keelung Rd., Taipei 106, Taiwan

Phone number: 886-2-33664173

E-mail: cmj@ntu.edu.tw

Table content

Table S1. PCR conditions……………………………………………………………………………………….2

Figure S1. Linear discriminant analysis (LDA) effect size of the OTUs with significant differences in abundance in rumen samples from regional farms. (A) Taxonomic cladogram of 16S rRNA sequences. (B) LDA scores of biomarkers in the rumen samples from different regions. The OTUs with LDA scores (log 10) higher than 4 were considered biomarkers for each group. Relative abundance of (C) Bacteroidaceae, Coriobacteriaceae, Lachnospiraceae, Methanobacteriaceae, Paraprevotellaceae, Prevotellaceae, Ruminococcaceae, Streptococcaceae, and Veillonellaceae…………………………………………………………………………………………………..….3

Table S1. PCR conditions

PCR condition	Temperature (ºC)	Time
Thermocycler	95	2 min
Denaturing	95	15 sec
Annealing	50	20 sec
Extension	68	45 sec
Amplification 12 cycles (Annealing temperature decrease 0.5 ºC every 1 cycle)		
Denaturing	95	15 sec
Annealing	44	20 sec
Extension	68	45 sec
Amplification 28 cycles		
Elongation	68	5 min

Figure S1.

(A)

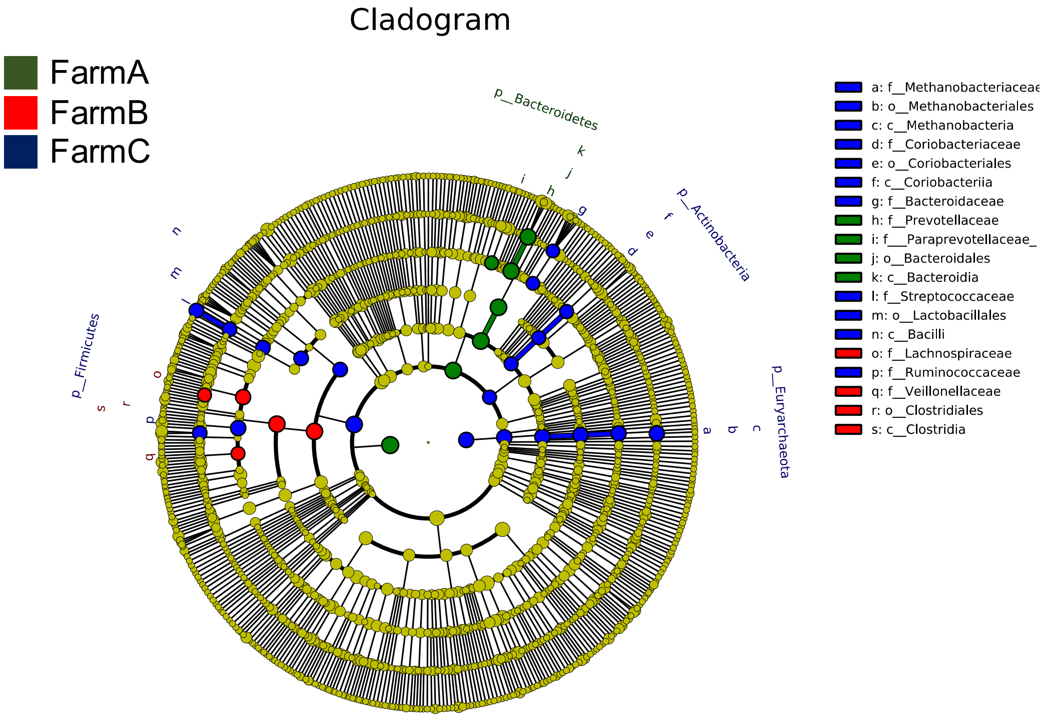
Cladogram
FarmA
FarmB
FarmC
p__Bacteroidetes
p__Actinobacteria
p__Euryarchaeota
p__Firmicutes
a: f__Methanobacteriaceae
b: o__Methanobacteriales
c: c__Methanobacteria
d: f__Coriobacteriaceae
e: o__Coriobacteriales
f: c__Coriobacteriia
g: f__Bacteroidaceae
h: f__Prevotellaceae
i: f___Paraprevotellaceae_
j: o__Bacteroidales
k: c__Bacteroidia
l: f__Streptococcaceae
m: o__Lactobacillales
n: c__Bacilli
o: f__Lachnospiraceae
p: f__Ruminococcaceae
q: f__Veillonellaceae
r: o__Clostridiales
s: c__Clostridia

(B)

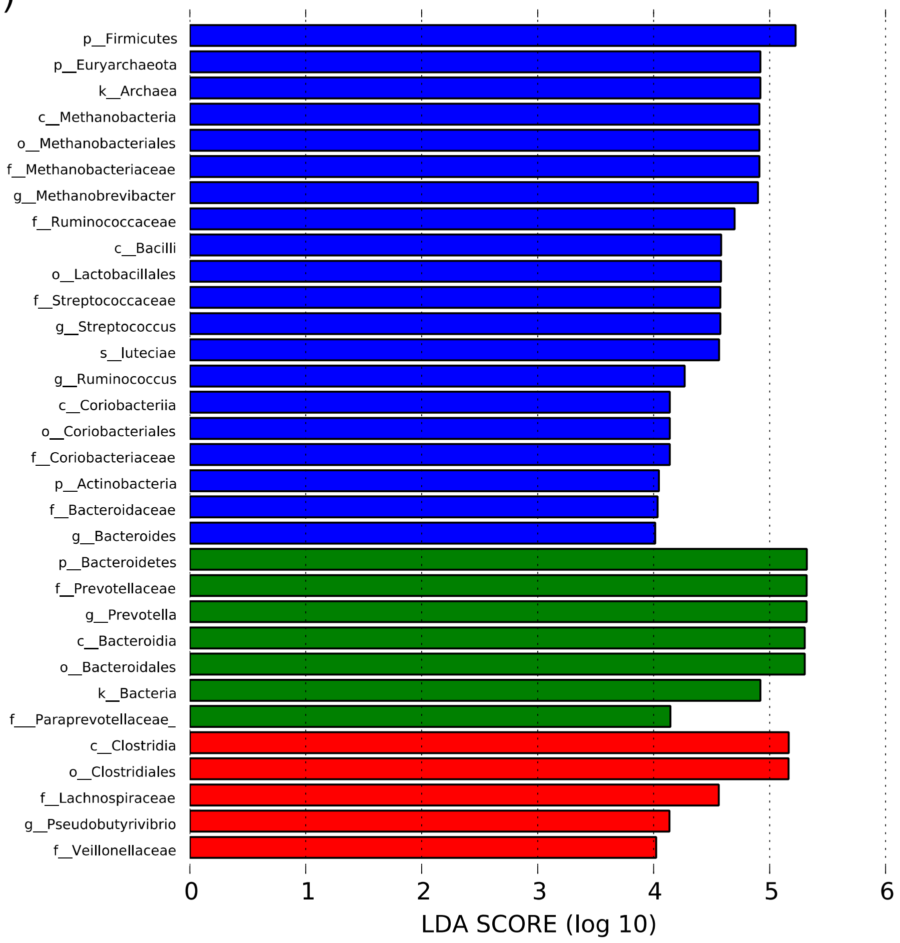
p__Firmicutes
p__Euryarchaeota
k__Archaea
c__Methanobacteria
o__Methanobacteriales
f__Methanobacteriaceae
g__Methanobrevibacter
f__Ruminococcaceae
c__Bacilli
o__Lactobacillales
f__Streptococcaceae
g__Streptococcus
s__luteciae
g__Ruminococcus
c__Coriobacteriia
o__Coriobacteriales
f__Coriobacteriaceae
p__Actinobacteria
f__Bacteroidaceae
g__Bacteroides
p__Bacteroidetes
f__Prevotellaceae
g__Prevotella
c__Bacteroidia
o__Bacteroidales
k__Bacteria
f___Paraprevotellaceae_
c__Clostridia
o__Clostridiales
f__Lachnospiraceae
g__Pseudobutyrivibrio
f__Veillonellaceae
0
1
2
3
4
5
6
LDA SCORE (log 10)

(C)

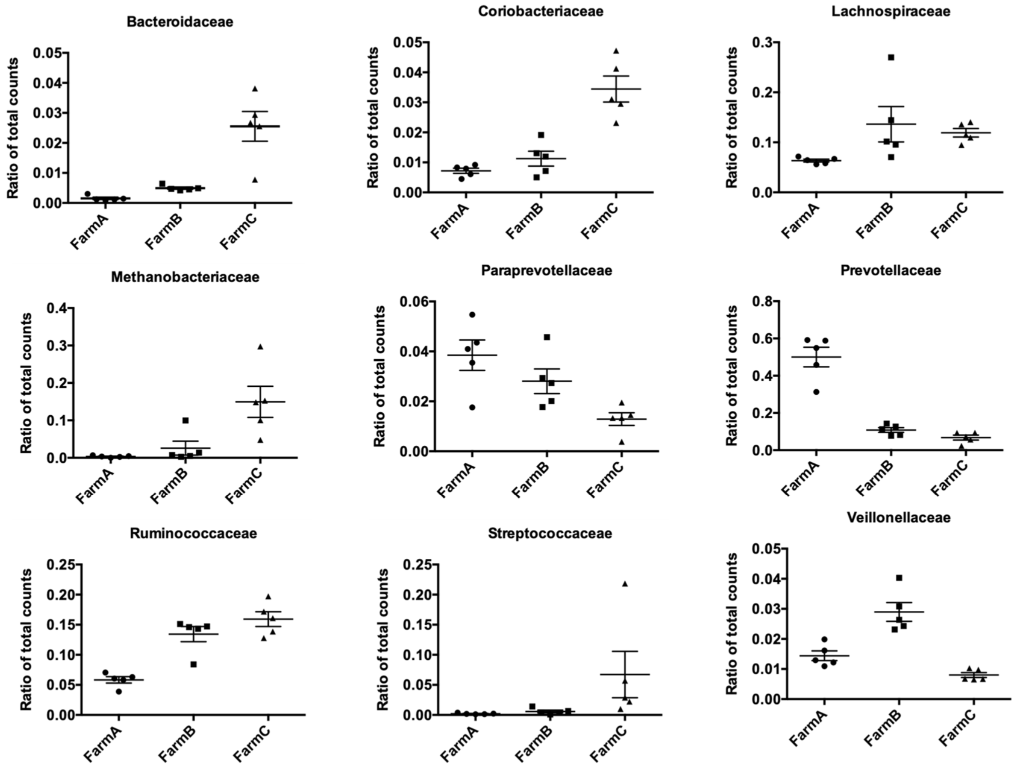

Figure S1. Linear discriminant analysis (LDA) effect size of the OTUs with significant differences in abundance in rumen samples from regional farms. (A) Taxonomic cladogram of 16S rRNA sequences. (B) LDA scores of biomarkers in the rumen samples from different regions. The OTUs with LDA scores (log 10) higher than 4 were considered biomarkers for each group. Relative abundance of (C) Bacteroidaceae, Coriobacteriaceae, Lachnospiraceae, Methanobacteriaceae, Paraprevotellaceae, Prevotellaceae, Ruminococcaceae, Streptococcaceae, and Veillonellaceae.
